# Extensive Calcific Chronic Pancreatitis Developing Within Three Years After Acute Pancreatitis and Incidentally Detected During Urologic Evaluation in a 34-Year-Old Man: A Case Report

**DOI:** 10.7759/cureus.113323

**Published:** 2026-07-24

**Authors:** Yoshihiro Kawaguchi, Kazuhiro Hamaoka

**Affiliations:** 1 Department of Urology, Saiseikai Futsukaichi Hospital, Chikushino, JPN; 2 Department of Urology, Morodomi Central Hospital, Saga, JPN; 3 Department of Gastroenterology, Morodomi Central Hospital, Saga, JPN

**Keywords:** acute pancreatitis, alcohol-related pancreatitis, chronic pancreatitis, kub radiograph, pancreatic calcification

## Abstract

Pancreatic calcification is a hallmark structural change of chronic pancreatitis. However, few reports have described extensive diffuse pancreatic calcification that develops within a relatively short period after acute pancreatitis and is incidentally detected during urological evaluations. We report the case of a 34-year-old man who presented with urinary frequency, gross hematuria, and the passage of sand-like material in his urine. He had smoked five cigarettes per day and consumed alcohol on approximately 10 occasions per month since the age of 29 years, when abnormal liver function was noted on routine tests, and he developed acute pancreatitis at the age of 31 years. Computed tomography (CT) and magnetic resonance cholangiopancreatography performed at that time revealed no pancreatic calcifications. At the current presentation, he was diagnosed with acute bacterial prostatitis, and a urine culture yielded *Escherichia coli*. A kidneys, ureters, and bladder (KUB) radiograph performed to evaluate suspected urolithiasis incidentally revealed multiple calcified densities in the pancreatic region. Moreover, noncontrast CT demonstrated extensive diffuse calcifications involving the entire pancreas, with mild pancreatic enlargement. Serum calcium, IgG4, intact parathyroid hormone, and HbA1c levels were within the normal ranges, suggesting that alcohol consumption and smoking were the most likely etiological factors. This case suggests that extensive calcific chronic pancreatitis can develop within a relatively short period following acute pancreatitis. Clinicians evaluating KUB radiographs and CTs performed for urological indications should systematically check for clinically significant incidental findings outside the urinary tract.

## Introduction

Chronic pancreatitis is a progressive inflammatory disease characterized by irreversible morphological changes in the pancreatic parenchyma and ductal system, resulting in exocrine and endocrine dysfunction. Pancreatic calcification is one of the most common structural changes and represents an important diagnostic feature of chronic pancreatitis [1,2].

It usually develops over several years through repeated pancreatic inflammation, eventually resulting in irreversible fibrosis and calcification. However, extensive calcification developing within only three years after an episode of acute pancreatitis is uncommon.

Alcohol consumption and smoking are major risk factors for chronic pancreatitis and may contribute to progressive pancreatic calcification even in young adults [3,4].

Few reports have described the development of extensive diffuse pancreatic calcifications within a relatively short period after acute pancreatitis, especially after no calcifications were detected on the initial computed tomography (CT) and magnetic resonance cholangiopancreatography (MRCP) images. We report a case of extensive calcific chronic pancreatitis that was incidentally detected during urological evaluation using kidneys, ureters, and bladder (KUB) radiography and CT in a 34-year-old man.

## Case presentation

A 34-year-old man presented to our urology department in the evening with urinary frequency, gross hematuria, and passage of sand-like material in his urine, which began earlier that morning. He had also experienced fever for three days before presentation.

The patient had smoked five cigarettes per day since the age of 29 years and consumed approximately 10 beers per drinking occasion, approximately 10 times per month. He also had abnormal liver function test results during routine health checkups since that time.

At the age of 31 years (2023), he was admitted to another hospital with acute pancreatitis. During the week preceding the onset of acute pancreatitis, he consumed approximately 10 glasses of highballs daily. Laboratory findings at that time showed an amylase level of 153 U/L, serum calcium of 9.5 mg/dL, IgG4 of 92.7 mg/dL, γ-glutamyl transferase (γ-GTP) of 195 U/L, a white blood cell count of 9,660/μL, and a C-reactive protein level of 0.05 mg/dL (Table 1).

**Table 1:** Comparison of laboratory findings obtained during the episode of acute pancreatitis in 2023 and at the current presentation. The table highlights changes in inflammatory markers and pancreatic enzymes, as well as laboratory investigations performed to evaluate the etiology of chronic calcific pancreatitis. HDL, high-density lipoprotein.

Parameter	Reference range	2023 (acute pancreatitis)	Current presentation
White blood cell count (/µL)	3500-9800	9660	14700
C-reactive protein (mg/dL)	0-0.30	0.05	9.89
Amylase (U/L)	42-132	153	57
Lipase (U/L)	13-55	Not measured	68
γ-Glutamyl transferase (U/L)	10-47	195	95
Calcium (mg/dL)	8.7-10.3	9.5	9
Triglycerides (mg/dL)	30-149	Not measured	177
HDL cholesterol (mg/dL)	40-96	Not measured	37
IgG4 (mg/dL)	11.0-121.0	92.7	92.9
HbA1c (%)	4.6-6.2	Not measured	5.7
Intact parathyroid hormone (pg/mL)	10-65	Not measured	58

**Figure 1:**
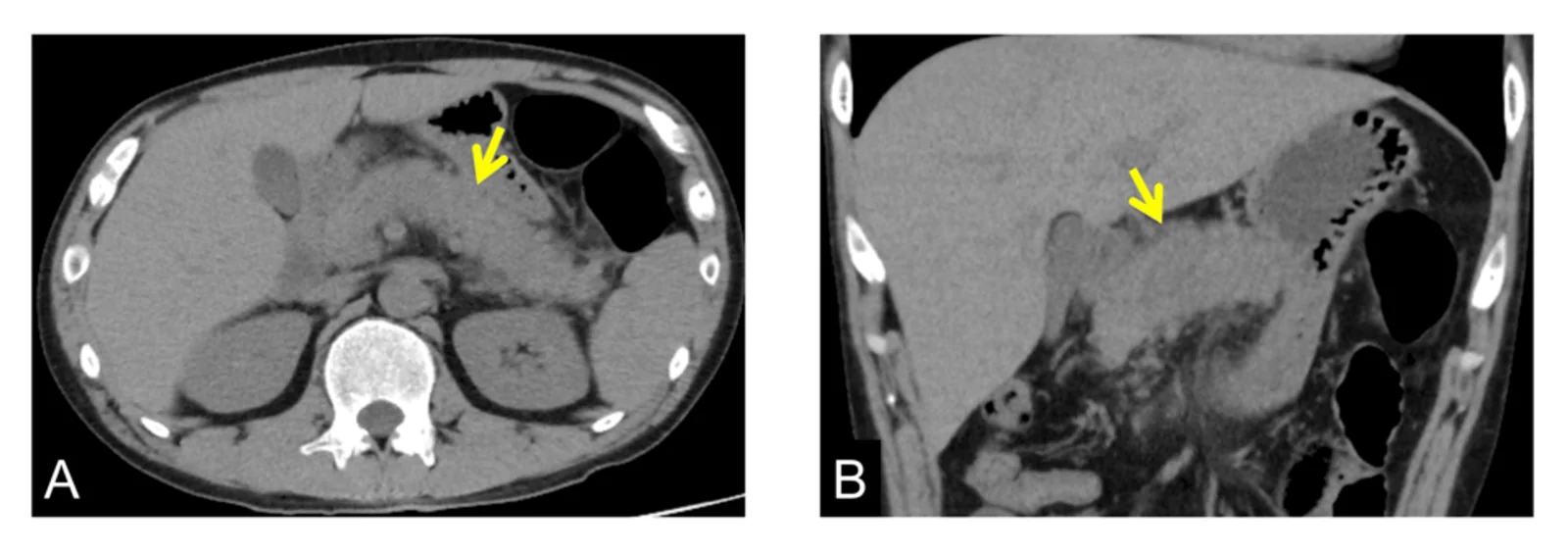
Noncontrast abdominal CT images obtained during the episode of acute pancreatitis in 2023. (A) Axial and (B) coronal views show no evidence of pancreatic calcification. The yellow arrow indicates the pancreas.

**Figure 2:**
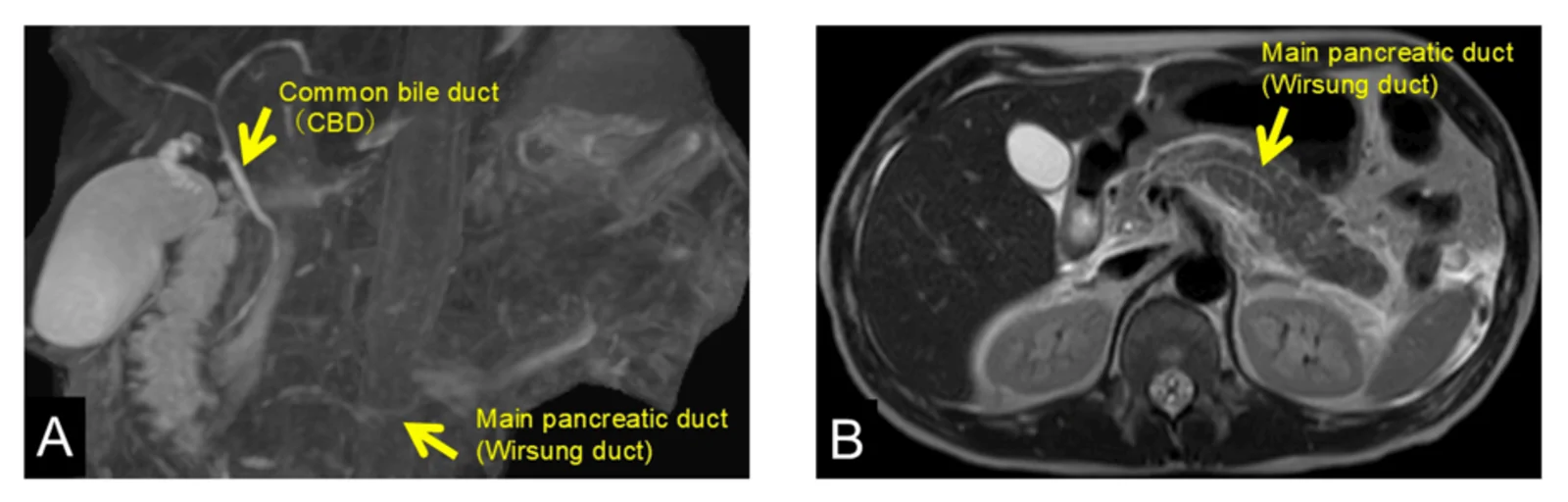
MRCP findings during the episode of acute pancreatitis in 2023. (A) Three-dimensional maximum intensity projection image and (B) axial T2-weighted image demonstrating no significant pancreatic duct dilatation or structural abnormalities. Yellow arrows indicate the main pancreatic duct (Wirsung duct) and the common bile duct (CBD). MRCP, magnetic resonance cholangiopancreatography.

CT and MRCP performed at that time revealed no pancreatic calcifications or apparent pancreatic duct abnormalities (Figures 1, 2).

At the current presentation, physical examination revealed no costovertebral angle tenderness but demonstrated perineal tenderness. Laboratory investigations showed a white blood cell count of 14,700/μL, a C-reactive protein level of 9.89 mg/dL, a serum lipase level of 68 U/L, triglycerides of 177 mg/dL, high-density lipoprotein cholesterol of 37 mg/dL, and γ-GTP of 95 U/L. Serum calcium, glycated hemoglobin (HbA1c), IgG, IgG4, and intact parathyroid hormone levels were all within normal ranges.

Urinalysis revealed marked pyuria, and urine culture yielded *Escherichia coli*, which was susceptible to all tested antimicrobial agents. The patient was diagnosed with acute bacterial prostatitis and treated as an outpatient with intravenous ceftriaxone (2 g/day) and fluid replacement therapy.

**Figure 3:**
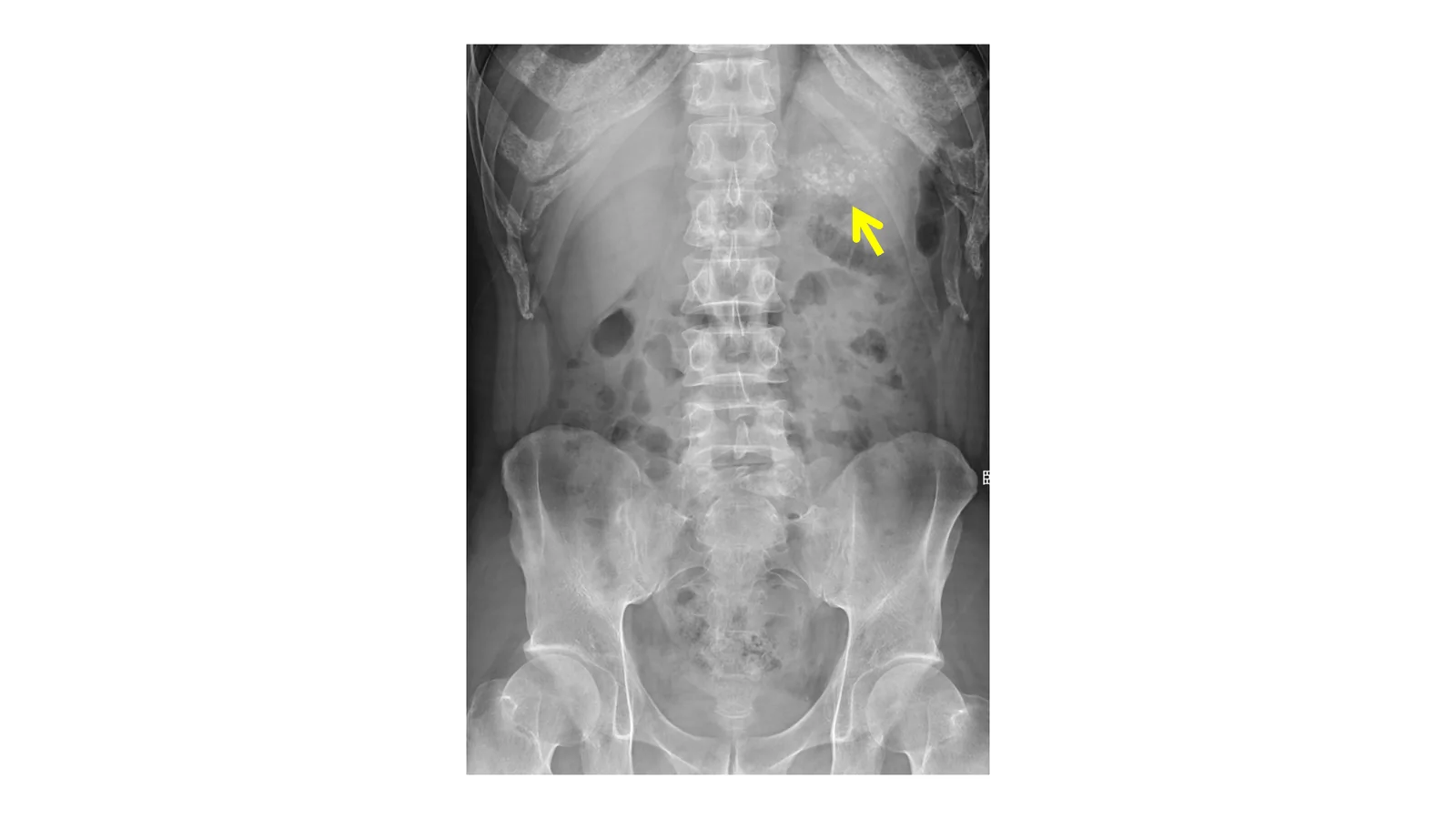
Incidental pancreatic calcifications on plain abdominal radiography. Plain abdominal radiography performed for suspected urolithiasis revealed multiple calcific densities in the pancreatic region, leading to an incidental diagnosis of extensively calcified chronic pancreatitis. The yellow arrow indicates pancreatic calcifications.

Plain abdominal radiography (KUB), performed to evaluate suspected urolithiasis, revealed multiple calcific densities corresponding to the pancreatic region (Figure 3).

**Figure 4:**
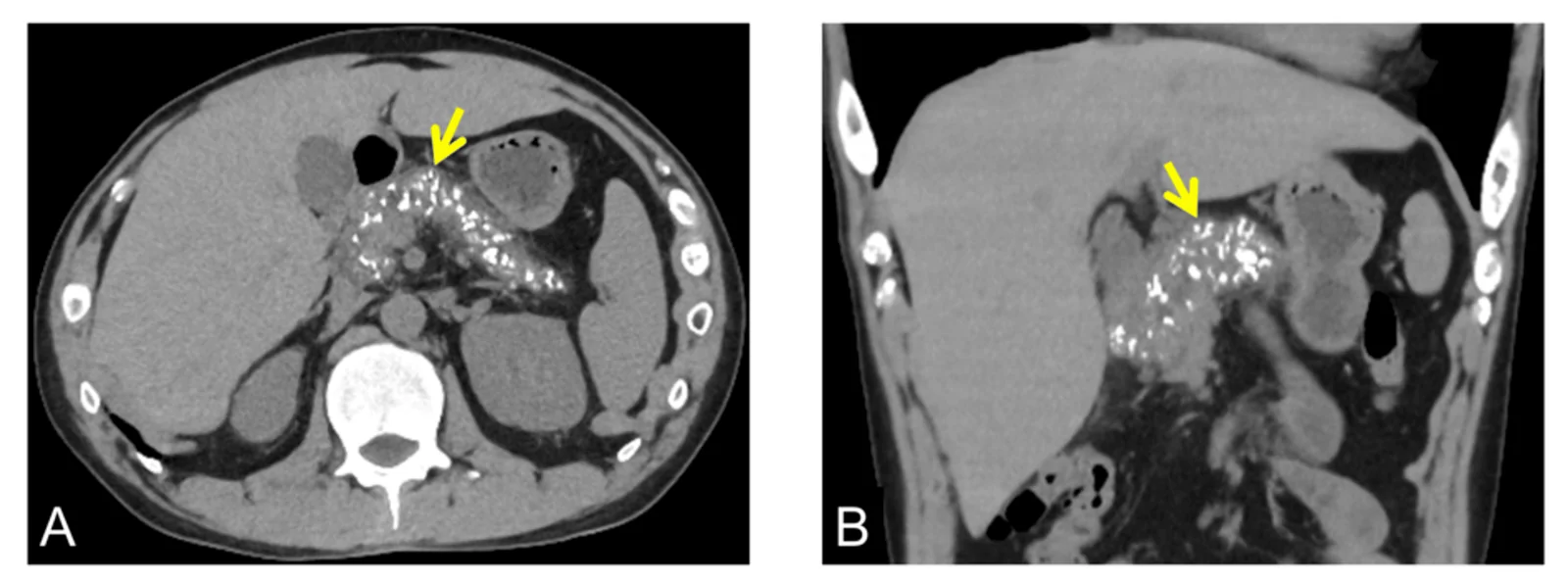
Extensive diffuse pancreatic calcifications on noncontrast CT. Noncontrast abdominal CT was performed at presentation. (A) Axial and (B) coronal views demonstrating extensive diffuse calcifications involving the entire pancreas, accompanied by mild pancreatic enlargement. No stones were detected in the urine. The yellow arrows indicate pancreatic calcifications.

Noncontrast CT revealed extensive diffuse pancreatic calcifications involving the entire pancreas, accompanied by mild pancreatic enlargement (Figure 4).

Following antimicrobial treatment, the patient’s inflammatory marker levels and urine findings improved by Day 5. He was subsequently switched to oral levofloxacin (500 mg/day), which was prescribed for a total treatment duration of four weeks. He is currently scheduled for a follow-up evaluation at a gastroenterology outpatient clinic.

## Discussion

This case report provides two important clinical lessons. First, extensive calcific chronic pancreatitis may develop within a relatively short period after acute pancreatitis. Second, clinically significant incidental findings outside the urinary tract should be systematically evaluated using KUB radiography and CT performed for urological indications.

Chronic pancreatitis is a progressive inflammatory disease characterized by irreversible changes in pancreatic parenchyma and ductal structures, with pancreatic calcification being one of the most characteristic structural abnormalities [1,2]. Pancreatic calcification is considered an important diagnostic feature of chronic pancreatitis even in the absence of overt clinical symptoms [2]. In the present case, neither CT nor MRCP performed at the time of acute pancreatitis at the age of 31 years demonstrated pancreatic calcifications, whereas extensive diffuse calcifications involving the entire pancreas were identified approximately three years later. This finding suggests that marked calcific changes may develop within a relatively short interval after acute pancreatitis.

Alcohol consumption and cigarette smoking are well-established risk factors for the development and progression of chronic pancreatitis [3,4]. Our patient had a history of smoking and heavy alcohol consumption since his late twenties, with abnormal liver function identified during routine health examinations. In addition, he had consumed approximately 10 glasses of highballs daily during the week preceding the onset of acute pancreatitis. In contrast, hypercalcemia, severe hypertriglyceridemia, and IgG4-related autoimmune pancreatitis were considered unlikely because the serum calcium, triglyceride, and IgG4 concentrations were within normal or near-normal ranges. Therefore, alcohol consumption and smoking were considered the most likely etiological factors contributing to the development of chronic calcific pancreatitis in this patient [1].

In the present case, pancreatic calcifications were incidentally identified on the KUB radiograph, and CT was performed for suspected urolithiasis. Previous reports have emphasized the clinical importance of recognizing incidental findings outside the primary target organs during abdominal imaging examinations [5,6]. Even in urological practice, physicians should systematically evaluate extraurinary structures using KUB radiography and CT to avoid overlooking clinically significant abnormalities. This case emphasizes the importance of a comprehensive image interpretation beyond the urinary tract.

Pancreatic calcification generally develops gradually during the progression of chronic pancreatitis and is typically associated with longstanding disease. Therefore, the development of extensive diffuse pancreatic calcification within approximately three years after an episode of acute pancreatitis in our patient appears rapid compared with the usual clinical course. Although the exact timing of calcification could not be determined, this case suggests that extensive calcification may develop earlier than generally expected in some patients. Furthermore, pancreatic exocrine function was not formally evaluated because fecal elastase or other exocrine function tests were not performed. Nutritional status was also not formally assessed. In addition, no evidence of overt endocrine dysfunction was identified based on the normal HbA1c level at the time of presentation. Therefore, subclinical pancreatic functional impairment could not be completely excluded.

This case has some limitations. First, endoscopic ultrasonography, endoscopic retrograde cholangiopancreatography, genetic testing, and evaluation for hereditary pancreatitis or pancreatic divisum were not performed, precluding the exclusion of these etiologies. Second, pancreatic exocrine function and nutritional status were not formally evaluated. Finally, because this is a single case report, a causal relationship between alcohol consumption, smoking, and the rapid development of pancreatic calcification cannot be established.

## Conclusions

This case suggests that extensive calcific chronic pancreatitis may develop within three years after acute pancreatitis, even when no calcifications are detected on initial CT or MRCP. Although alcohol consumption and smoking may have contributed to disease progression, a causal relationship cannot be established based on a single case. Careful evaluation of extraurinary structures on abdominal imaging may facilitate the detection of clinically significant incidental findings. This case may contribute to the understanding of the radiological progression of chronic pancreatitis; however, larger studies are needed to clarify the frequency and mechanisms of rapid pancreatic calcification.
